# Distress Profiles of Adolescents with Gender Dysphoria: A Cluster Analysis Approach

**DOI:** 10.1007/s10508-025-03221-3

**Published:** 2025-08-20

**Authors:** André Leonhardt, Martin Fuchs, Gabriele Kohlboeck, Nora Bachler-Ortner, Nina Haid-Stecher, Manuela Gander, Kathrin Sevecke

**Affiliations:** 1 Institute of Psychology, University of Innsbruck, Universitätsstraße 15, 6020 Innsbruck, Austria; 2 Department for Child and Adolescent Psychiatry, Medical University of Innsbruck, 6020 Innsbruck, Austria; 3 Department for Child and Adolescent Psychiatry, Psychotherapy and Psychosomatics, Tirol Kliniken, Milser Straße 10, 6060 Hall in Tirol, Austria

**Keywords:** Gender dysphoria, Adolescents, Psychopathology, Personality functioning, Childhood trauma

## Abstract

Gender dysphoric adolescents presenting to specialized gender identity services experience varying degrees of co-occurring mental health problems. However, few studies have examined how these symptoms may cluster into distinct patterns of psychological distress. This study examined subgroup differences in psychopathological characteristics in a clinical sample of 102 adolescents (74.5% natal females, M age = 16.08 years, SD = 1.54) with gender dysphoria. We used hierarchical cluster analysis to identify subgroups within our sample based on self-report measures on gender dysphoria, general psychopathology, body image, childhood trauma, levels of personality functioning, identity development, and items on perceived social support. This exploratory approach revealed three subgroups with distinct distress profiles. A “Low-Distress” cluster (29%) showed minimal psychopathological symptoms, with scores below clinical cut-offs on most measures and high levels of social support. A “Moderate-Distress” cluster (48%) showed significant internalizing psychopathology, moderate exposure to emotional maltreatment, and low to moderate impairment in personality functioning, but high levels of social support. A “High-Distress” cluster (23%) showed severe internalizing and externalizing psychopathology, high levels of childhood trauma on most subscales, severe impairments in personality functioning and identity development and low levels of social support. All clusters reported clinically significant gender dysphoria and poor body image. These findings highlight significant differences in distress profiles in this clinical sample and provide a basis for future research to validate this subgroup typology, examine their developmental trajectories, and inform individualized treatment approaches for adolescents with gender dysphoria.

**Supplementary Information:**

The online version contains supplementary material available at 10.1007/s10508-025-03221-3.

## Introduction

Over the past 10–15 years, there has been a notable increase in the number of adolescents presenting to specialized gender identity services for treatment of an incongruence between their experienced gender identity and their natal sex (Thompson et al., 2022a). The resulting mental distress is diagnosed as Gender Dysphoria according to the fifth edition of the *Diagnostic and Statistical Manual of Mental Disorders* (DSM-5; American Psychiatric Association [APA], 2013). Many of these adolescents express a desire for body modification and seek hormonal or surgical treatment, with a marked increase in natal female adolescents compared to natal males (Aitken et al., 2015; Taylor et al., 2024). The rise in the number of minors seeking treatment, combined with the shift in the sex ratio favoring natal females, has given rise to contentious debates concerning the underlying factors for these developments and appropriate clinical management (Levine & Abbruzzese, 2023; Zucker, 2019).

Gender dysphoric adolescents attending clinical services often report high levels of distress, comparable to those observed in adolescents referred to mental health services for other reasons (Kaltiala-Heino et al., 2018; Thompson et al., 2022b; Zucker, 2019). While previous research mostly focused on individual aspects of mental health or developmental characteristics (Thompson et al., 2022b), few studies have examined clinical cohorts across multiple domains to identify potential subgroups with distinct constellations of psychopathological characteristics. A descriptive differentiation of subgroups and the subsequent definition of typologies for individuals with gender dysphoria have been established for adults, focusing on sexual orientation or age of onset (Lawrence, 2010). Typologies are intended to provide concise clinical descriptions and to help target interventions to specific needs. They can also facilitate research on developmental trajectories to promote the development of prognostic models. Our study follows this tradition and implements a novel approach. We aim to identify subgroups within a clinical cohort of gender dysphoric adolescents on the basis of their psychopathological characteristics and derive an initial proposal for a possible typology based on distress profiles.

By introducing such a typology, the goal is to provide a framework that contributes to a deeper understanding of the patterns of psychopathological impairments and developmental characteristics within this population. Another aim is to contribute to the existing literature by examining a number of understudied variables of considerable clinical and developmental importance, such as childhood trauma, personality functioning, and identity development, while also considering general psychopathology and body image. We also collected data on self-reported onset of gender dysphoria. As noted in a systematic review by Thompson et al. (2022a), age of onset is rarely reported in the adolescent literature, making this study one of the few to provide such data. We also included items measuring perceived social support. Research has highlighted the importance of social support in reducing psychopathology in transgender youth (Durwood et al., 2021). Its clinical importance is further underscored by the fact that some international guidelines require family/social support for medical treatment (Cass, 2024).

Poor body image has been shown to be significantly higher in gender dysphoric adolescents compared to population-based norms (Becker et al., 2018) and a study by Herrmann et al. (2023) found that 70% of a clinical sample of 114 gender dysphoric adolescents identified body discomfort as a key factor influencing their gender development and gender experience. Poor body image can be understood as a symptom commonly associated with gender dysphoria. For some individuals it may also reflect developmental struggles that manifest at the level of the body, which then serves as a site of psychodynamic conflict, causing discomfort (Lemma, 2021). These processes are not mutually exclusive and may interact in complex ways that warrant further investigation.

A high prevalence of childhood trauma and adverse childhood experiences (ACEs) in gender dysphoric adolescents has so far mostly been inferred from studies with clinical samples of adults (Biedermann et al., 2021; Feil et al., 2023; Giovanardi et al., 2018). While Feil et al. included a non-clinical comparison group, the other studies focused on clinical cohorts. In one of the few existing studies with gender dysphoric adolescents, Kozlowska et al. (2021a) examined rates of unresolved loss/trauma in 57 children and adolescents presenting to a specialist service. This clinical cohort reported significantly higher levels of various forms of trauma—including physical and psychological abuse, neglect, and loss of parental figures—compared to a non-clinical control group whereas no significant differences were found when compared with a mixed psychiatric control group. Another study also found high rates of ACEs in a clinical cohort of gender dysphoric youth (Kozlowska et al., 2021b). Such traumatic childhood experiences can have a detrimental impact on personality and identity formation and disrupt the development of a coherent sense of self during adolescence (Fonagy et al., 2002; Schore, 2003).

Although these associations of childhood trauma with personality functioning and identity development are well documented, these aspects remain understudied in gender dysphoric youth. To date, two studies have assessed identity integration in gender dysphoric adolescents using the Assessment of Identity Development in Adolescence (AIDA; Goth et al., 2012), a self-report questionnaire designed to discern between healthy identity development, transient identity crises, and identity diffusion. Identity diffusion describes the inability to form a coherent and continuous sense of self, is characterized by a fragmented and unstable concept of the self and significant others, and is conceptualized as the basis for subsequent personality pathology (Gander et al., 2025; Goth et al., 2012; Kernberg, 1986). Haid-Stecher et al. (2020) found that 36% of gender dysphoric adolescents referred to a specialized gender identity service showed clinically significant identity diffusion, while the sample as a whole scored within the average range compared to normative data. Karvonen et al. (2022) on the other hand observed no significant differences in overall identity development between gender dysphoric adolescents and the general population, highlighting the heterogeneity within the limited data on identity diffusion in this clinical population.

This brief review of research on body image, childhood trauma, personality functioning, and identity development in gender dysphoric youth highlights the need for further research to gain a more comprehensive understanding of the developmental challenges of gender dysphoric adolescents. While previous research has mostly examined these factors individually, little is known about how they co-occur and interact in adolescents with gender dysphoria. In addition, variables such as the onset of gender dysphoria and the role of social support may shape psychosocial experiences and further contribute to subgroup differentiation.

### Aim of This Study

Building on previous research on subgroup differences and typologies in individuals with gender dysphoria, our study aimed to examine a cohort of gender dysphoric adolescents referred to a gender identity service with regard to a range of psychopathological variables, including general psychopathology, body image problems, childhood trauma, personality functioning, and identity development. We applied the statistical method of hierarchical cluster analysis to a comprehensive collection of diagnostic data, including variables that have received limited attention in previous research. Based on this approach, we proposed a distress-based typology for adolescents with gender dysphoria as a potential descriptive framework for this heterogeneous population and discussed its potential implications for clinical management and future research.

## Method

### Participants

A total of 198 adolescents attended at least one initial clinical consultation at a specialized gender identity service. Of these, 193 were between the ages of 12 and 18, and 151 (78.2%) completed the questionnaire-based assessment. For the present analysis, inclusion was restricted to participants with complete data on all self-report measures. Several measures were added after the initial data collection began. As a result, 49 participants with missing data on key measures were excluded from the analysis. A total of 42 adolescents did not complete any questionnaire-based assessments after their initial clinical consultation, and no data were available for these cases. Thus, the final sample consisted of 102 adolescents (74.5% natal females) with complete data on all variables included in the analysis and whose parents consented to their participation in the study. A comparison between the 102 included and the 49 excluded adolescents on demographic characteristics and Youth Self-Report (YSR) scores is presented in Supplementary Table S1. All participants included in the final sample were clinically diagnosed with gender dysphoria according to DSM-5 criteria by experienced professionals prior to participation. All data were collected shortly after the initial clinical consultation and diagnostic assessment.

### Procedure

This study was part of the Tyrolean Transgender Study in Austria aimed to investigate the mental health and developmental trajectories of children and adolescents with gender dysphoria. All participants sought treatment at the specialized gender identity service at the Department of Child and Adolescent Psychiatry at the Medical University Innsbruck, Austria, for a psychiatric assessment and treatment between December 2017 and March 2024. Eligibility criteria were residence in Austria or South Tyrol (Italy), knowledge of the German language, and the cognitive ability to complete an online questionnaire.

### Measures

With the exception of one measure of social support, all of the questionnaires used in this study have demonstrated solid psychometric properties in previous research, confirming their reliability and validity for use in this study. In addition, internal consistency analyses were conducted for the present study and are detailed in each section.

#### Sociodemographic Characteristics, Reported Onset of Gender Dysphoria and Sexual Orientation

These included age, natal sex, educational attainment, employment status, parental marital status, and living arrangements.

As part of the medical history, patients were asked to report the age at which they first experienced discomfort with their gender ("At what age did you first notice feeling discomfort with your gender?"). Responses indicating an onset before the age of 10 were classified as early onset. For example, answers such as “since kindergarten” or “always” were classified as < 10 years and therefore as early onset. Onset at age 10 or older was classified as late onset. These two categories reflect the most commonly differentiated developmental pathways in the literature on gender dysphoria (Zucker, 2019).

Participants were also asked to report their sexual orientation using a single item from the Sexual Experiences and Sexual Orientation among Transgender Adolescents questionnaire (Stübler & Becker-Hebly, 2019): "Who are you sexually attracted to?" Response options were: “To no one,” “to girls,” “more to girls, sometimes also to boys,” “to both,” “more to boys, sometimes also to girls,” “to boys,” and “other.” For reporting purposes, responses were grouped into two categories based on natal sex: same-sex or both-sex attracted, and all other orientations (including opposite-sex attraction, no attraction, ambiguous attraction, and “other”).

#### Gender Dysphoria

The Utrecht Gender Dysphoria Scale (UGDS; Cohen-Kettenis & Van Goozen, 1997) is a 12-item self-report questionnaire that assesses gender dysphoria by measuring distress related to body dissatisfaction and gender role discomfort. The UGDS is available in male and female versions, administered according to natal sex. Items such as “Every time someone treats me like a girl/boy, I feel hurt” or “I hate my body” were rated on a 5-point scale ranging from 1 (“Disagree completely”) to 5 (“Agree completely”). A total score was calculated, with higher scores indicating more severe gender dysphoria. In the original study (Cohen-Kettenis & van Goozen, 1997), Cronbach’s α was reported as 0.92 for the male version and 0.78 for the female version. A validation study by Steensma et al. (2013) reported Cronbach’s α = 0.98 for both versions. The UGDS has also been evaluated in comparative clinical and community-based samples (Galupo & Pulice-Farrow, 2020; Schneider et al., 2016). In the present study, internal consistency was high (female version: α = 0.88; male version: α = 0.94).

The Gender Identity/Gender Dysphoria Questionnaire for Adolescents and Adults (GIDYQ-AA; Deogracias et al., 2007) is a 27-item self-report questionnaire that measures gender identity and gender dysphoria over the past 12 months. The questionnaire is available in a male and female version and was completed according to the participant’s natal sex. Items such as “Have you felt more like a boy/girl than a girl/boy in the last 12 months?” were rated on a 5-point scale ranging from 1 (“Always”) to 5 (“Never”), with some items reverse-scored. A mean total score was calculated, with lower scores indicating greater gender dysphoria. Scores below 3.0 suggest clinically significant gender dysphoria. Deogracias et al. (2007) reported excellent internal consistency (Cronbach’s α = 0.97). Further validation was provided by Singh et al. (2010). The GIDYQ-AA has also been evaluated in comparative clinical and community-based samples (Galupo & Pulice-Farrow, 2020; Schneider et al., 2016). In the present study, the female version of the GIDYQ-AA showed acceptable internal consistency (α = 0.76), while the male version showed lower reliability (Cronbach's α = 0.52). Results of the male version should therefore be interpreted with caution.

#### Social Support

Perceived social support was measured using five items developed for clinical assessment in a gender identity service setting. The items captured participants’ subjective experiences of support in relation to gender dysphoria and gender transition. Participants rated the following items: support from others in transitioning (yes/no), parental support in transitioning (1 = no support to 5 = strong support), openness in discussing transition with parents (1 = never to 5 = always), feeling understood and accepted by their parents as they are (1 = never to 5 = always), and any social problems due to their gender identity (e.g., friends, school; 1 = never to 5 = always). Each item was analyzed individually. No total score was calculated.

#### General Psychopathology

The German version of the Youth Self-Report (YSR; Achenbach, 1991; Döpfner et al., 2014) is a 112-item self-report questionnaire assessing mental health problems in the past 6 months in adolescents aged 11 to 18. Items were rated on a 3-point scale ranging from 0 (“Not true”), 1 (“Somewhat or sometimes true”), to 2 (“Very true or often true”). Raw scores for each of the eight problem scales were converted into *T*-scores based on age- and sex-specific norms from the German standardization sample (Döpfner et al., 2014). Higher *T*-scores indicate greater symptom severity, with scores above 60 considered clinically significant. The YSR provides scores for eight problem scales: Anxious/Depressed, Withdrawn/Depressed, Somatic Complaints, Social Problems, Thought Problems, Attention Problems, Rule-Breaking Behavior, and Aggressive Behavior. Cronbach's α ranging from 0.56 to 0.86 have been reported for the internal consistency of the eight syndrome scales (Döpfner et al., 2014). In the present study, internal consistency was acceptable to high across all subscales: Anxious/Depressed (α = 0.87), Withdrawn/Depressed (α = 0.83), Somatic Complaints (α = 0.80), Social Problems (α = 0.69), Thought Problems (α = 0.84), Attention Problems (α = 0.77), Aggressive Behavior (α = 0.83), and Rule-Breaking Behavior (α = 0.74).

#### Body Image

The Body Image Scale (BIS; Lindgren & Pauly, 1975) is a 30-item self-report questionnaire, with each item assessing satisfaction with a particular body part. Items were rated on a 5-point scale ranging from 1 (“Very satisfied”) to 5 (“Very dissatisfied”). A total score was calculated, with higher scores indicating greater body dissatisfaction. In addition, a subscale score for neutral (non–sex-related) body parts was computed. The BIS has demonstrated excellent internal consistency in previous studies, with Cronbach’s α ranging from 0.91 to 0.93 (Brecht et al., 2024), and is considered age-appropriate for individuals aged 12 years and older. In the present study, internal consistency was high for both versions: female (α = 0.86), male (α = 0.83).

The Fragebogen zur Beurteilung des eigenen Körpers (FBeK; Strauß & Richter-Appelt, 1996) is a 52-item self-report questionnaire measuring body-related self-perception in individuals aged 12 years and older. Items were rated on a 2-point scale (1 = “Agree”, 2 = “Disagree”). The FBeK comprises four scales: Attractiveness and Self-Confidence (reverse-scored; e.g., “I am satisfied with my appearance”), Accentuation of Physical Appearance (e.g., “I frequently look in the mirror”), Insecurities and Concerns Regarding Physical Processes (reverse-scored; e.g., “My body often does what it wants”), and Physical-Sexual Discomfort (e.g., “I am satisfied with my sexual experience”). Higher scores indicate greater difficulties in the respective domain. The questionnaire has demonstrated acceptable to good internal consistency, with a Cronbach’s α between 0.69 and 0.85 (Brähler et al., 2000). In this study, reliability varied across subscales and for the respective version according to natal sex: Attractiveness and Self-Confidence (female: α = 0.79, male: α = 0.70), Accentuation of Physical Appearance (female: α = 0.55, male: α = 0.58), Insecurities and Concerns (female: α = 0.73, male: α = 0.70), and Physical-Sexual Discomfort (female: α = 0.29, male: α = 0.34). Given the low internal consistency of the Physical-Sexual Discomfort and Accentuation of Physical Appearance scales, results should be interpreted with caution.

#### Childhood Trauma

The Childhood Trauma Questionnaire (CTQ, Bernstein & Fink, 1998) is a 28-item self-report questionnaire that assesses childhood trauma retrospectively. The questionnaire consists of five subscales: Physical Abuse, Physical Neglect, Emotional Abuse, Emotional Neglect, and Sexual Abuse. Items were rated on a 5-point scale ranging from 1 (“Never true”) to 5 (“Very often true”), with some items reverse-scored. Each subscale results in a score between 5 and 25, with higher scores indicating greater exposure to traumatic experiences. Bernstein and Fink (1998) established cut-off scores for none, mild, moderate, and severe exposure levels. Moderate to severe trauma exposure is defined as scores of ≥ 13 for Emotional Abuse, ≥ 15 for Emotional Neglect, ≥ 10 for Physical Abuse, ≥ 10 for Physical Neglect, and ≥ 8 for Sexual Abuse. The German version of the CTQ has demonstrated good reliability and validity scores in a large psychiatric and community-based sample, with internal consistency exceeding α = 0.80 for most subscales (Klinitzke et al., 2011). In the present study, internal consistency for the CTQ subscales was high to excellent: Emotional Abuse (α = 0.88), Physical Abuse (α = 0.85), Sexual Abuse (α = 0.95), Emotional Neglect (α = 0.90), and Physical Neglect (α = 0.76).

#### Personality Functioning

The Levels of Personality Functioning Questionnaire (LoPF-Q 12–18; Goth et al., 2018) is a 97-item self-report questionnaire for adolescents aged 12–18 years that assesses four dimensions of personality functioning (Identity, Self-Direction, Empathy, and Intimacy) based on the DSM-5 Alternative Model for Personality Disorders (AMPD; APA, 2013) and the eleventh edition of the *International Classification of Diseases* (ICD-11; WHO, 2019). Items such as “I am confused about what kind of person I really am” (Identity) and “I often don’t understand other people’s reactions to my behavior” (Empathy) were rated on a 5-point scale ranging from 0 (“No”) to 4 (“Yes”), with intermediate options 1 (“Rather no”), 2 (“Partly”), and 3 (“Rather yes”). Higher scores indicate greater impairment in personality functioning. *T*-scores were calculated based on age- and sex-specific norms from the German standardization sample. *T*-scores below 60 are considered within the normal range, scores between 61 and 70 indicate mild to moderate impairment, and scores above 70 suggest severe impairment. The German version has demonstrated good scale reliability, with Cronbach´s α = 0.97 for the total score and α = 0.92, 0.94, 0.87, and 0.92 for the primary scales (Goth et al., 2018). Internal consistency for this study was high to excellent across all subscales: Identity (α = 0.88), Self-Direction (α = 0.93), Empathy (α = 0.90), and Intimacy (α = 0.91).

#### Identity Diffusion

The Assessment of Identity Development in Adolescence (AIDA; Goth et al., 2012) is a 58-item self-report questionnaire for adolescents aged 12–18 that assesses identity development in line with the Criterion A domain of the DSM-5 AMPD (APA, 2013). The AIDA distinguishes between healthy identity development, identity crisis, and identity diffusion. Items such as “I am confused about what kind of person I really am” were rated on a 5-point scale ranging from 0 (“No”) to 4 (“Yes”), with intermediate options 1 (“Rather no”), 2 (“Partly”), and 3 (“Rather yes”). A total Identity Diffusion score was calculated by summing all 58 items and converting the result into a *T*-score. Higher scores reflect greater identity diffusion. A *T*-score below 60 is considered within the normal range, scores between 61 and 70 indicate mild to moderate impairment, and scores above 70 indicate severe impairment. The questionnaire has demonstrated strong psychometric properties, with excellent reliability for the total Identity Diffusion scale (Cronbach’s α = 0.94). Internal consistency in the present study was excellent (α = 0.95).

### Data Analysis

Only participants with complete data on all included questionnaire measures and demographic variables were included in the analysis. We used exploratory data analysis methods to identify subgroups (i.e., clusters) of adolescents with similar characteristics in the dataset. We first performed a factor analysis of mixed data (FAMD), a principal component analysis (PCA) for quantitative and qualitative variables using the R package FactoMineR (https://cran.r-project.org/package=FactoMineR), and the factoextra package (https://cran.r-project.org/package=factoextra) to extract the FAMD results.

FAMD is a method similar to PCA and is often used as a data preprocessing step prior to further analysis. It can be applied to both numeric and categorical data, making it a useful tool for mixed data types (Husson et al., 2010; Lê et al., 2008). FAMD scales numerical variables and converts categorical variables into binary indicators, balancing their influence in the analysis. We included 34 demographic and clinical variables, resulting in a high variable-to-sample ratio relative to our sample of 102 participants. FAMD extractes latent factors, capturing the main structure and variance in the data, thereby reducing dimensionality and mitigating multicollinearity (Mahmood, 2021). This makes it suitable for small samples, since it helps summarize key relationships across variables while minimizing the risk of overfitting (Lê et al., 2008; Pagès, 2004). This preprocessing step enables stable and interpretable clustering solutions (Husson et al., 2010; Mahmood, 2021).

We applied hierarchical clustering on the principal components (HCPC) derived from FAMD using the HCPC function in the FactoMineR package, which enabled the identification of homogeneous groups based on mixed-type data (Husson et al., 2010). Using Ward’s method and Euclidean distance, this approach minimized within-cluster variance at each step (Husson et al., 2010; Murtagh & Contreras, 2012). To determine the optimal number of clusters, we used the NbClust package, which compares clustering solutions based on multiple indices (Charrad et al., 2014).

Hierarchical clustering was well suited for our small sample sizes and exploratory analyses because it does not rely on probabilistic assumptions and does not require a pre-determined number of clusters (Hastie et al., 2009; Kaufman & Rousseeuw, 1990; Lê et al., 2008). Based on pairwise distances, it can reveal nested structures and meaningful relationships even in low-power datasets.

The two-step approach combining FAMD and hierarchical clustering allowed us to extract factors that represent the core structure of the data and to identify homogeneous clusters based on these components. This approach improved interpretability in small samples and helps avoid overfitting (Kaufman & Rousseeuw, 1990; Lê et al., 2008; Pagès, 2004).

Group differences and relationships between variables at the level of nominal data were examined using Pearson's chi-squared tests. Where chi-squared tests did not fit the data, Fisher's exact probability test was used. One-way analysis of variance (ANOVA) was used to compare differences between clusters for continuous variables, and Pearson's chi-square test was used to assess differences between categorical variables; means and SDs were used to describe continuous variables. Multiple comparison tests (Scheffé post-hoc tests for equal variances and Games-Howell for unequal variances) were used to determine which specific clusters differed from each other (pairwise comparisons).

The statistical software package R Studio (R Studio version 2022.07.2, 2022) was used for FAMD, hierarchical clustering on principal components, and statistical sensitivity analysis. The Statistical Package for the Social Sciences (SPSS; version 29) was used to perform descriptive and inferential analyses (e.g., *t* tests, one-way ANOVA, post hoc Scheffé tests, chi-square tests, logistic regressions) to examine group differences, associations, and predictors. Zero-order correlations were calculated using SPSS and are presented in the supplementary material. A sensitivity analysis (*n* = 102, power = 0.80, α = 0.05) indicated that the minimum detectable effect size was Cohen’s *d* = 0.44, corresponding to a small-to-medium effect. While this level of sensitivity allowed for the reliable detection of medium-sized effects, smaller effects were more difficult to detect with the current sample size.

## Results

### Descriptive Data and Sociodemographic Characteristics

Table 1 shows the sociodemographic characteristics of the final sample, divided by natal sex. Pairwise comparisons were conducted to examine sex differences. No significant differences were observed for most characteristics, including age of onset of gender dysphoria and sexual orientation.

**Table 1:** Sociodemographic characteristics of the sample by natal sex

	Female *N* = 76	Male *N* = 26	*p*
Age in years (mean, SD)	15.9 (1.4)	16.5 (1.8)	0.121
Living with mother			
Biological mother^a^	68 (94.4%)^a^	25 (96.2%)	0.462
Not living with Biological mother (i.e. Adoptive mother, Foster mother, Stepmother; Replacement mother)	4 (5.5%)	1 (3.8%)	
Living with father^b^			0.570
Biological father	40 (54.8%)^b^	15 (57.7%)	
Not living with biological mother (i.e. adoptive father, foster father, step father; replacement father)	33 (45.2%)	11 (42.3%)	
Marital status of parents			
Separated/divorced	31 (40.8%)	10 (38.5%)	0.661
Deceased	2 (2.6%)	0 (0%)	
Never lived together	2 (2.6%)	0 (0%)	
Married partnership	41 (53.9%)	16 (61.5%)	
Place of residence:			
City > 100,000 inhabitants	14 (18.4%)	7 (26.9%)	0.479
Rural	36 (47.4%)	13 (50.0%)	
Small town	26 (34.2%)	6 (23.1%)	
Occupation			
Apprenticeship/work	12 (15.8%)	3 (11.5%)	0.914
Unemployed or looking for a job	9 (11.8%)	3 (11.5%)	
Attending school	51 (67.1%)	19 (73.1%)	
School drop out	1 (1.3%)	0	
Vocational training	3 (3.9%)	1 (3.8%)	
Onset of gender dysphoria			0.208
Early (≤ 10 years)	43 (56.6%)	11 (42.3%)	
Late (> 10 years)	33 (43.4%)	15 (57.7%)	
Sexual orientation^c^			
Same-sex or both-sex attracted	53 (72.6%)	17 (68.0%)	0.660
Other	20 (27.4%)	8 (32.0%)	

^a^Responses for living with mother based on 72 females, 26 males

^b^Responses for living with father based on 73 females, 26 males

^c^Responses for sexual orientation based on 73 females, 26 males

Response rates varied slightly across these variables due to partial nonresponse

### Factor Analysis of Mixed Data and Hierarchical Cluster Analysis (2-Step Approach)

**Fig. 1:**
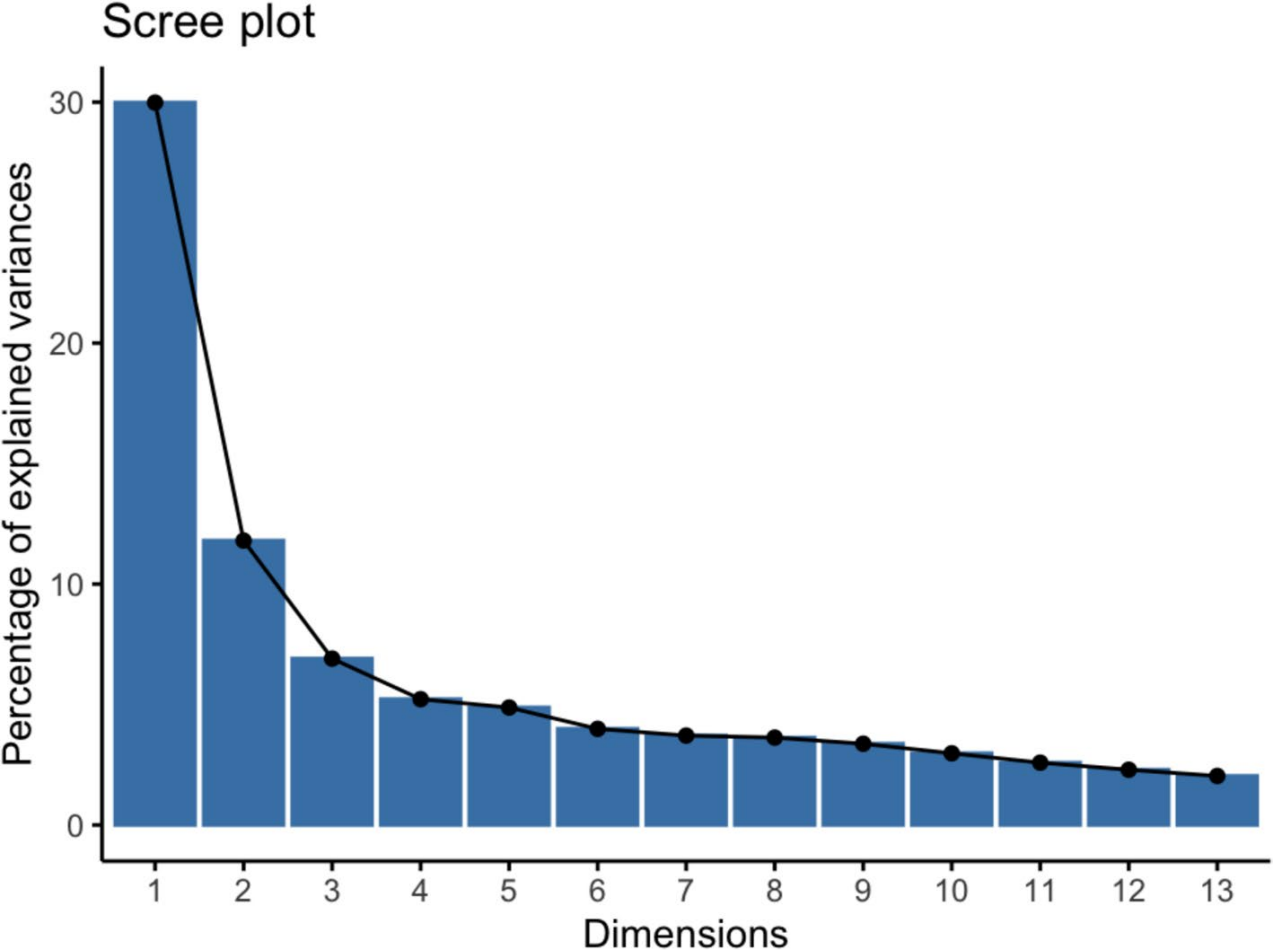
Scree plot showing the percentage of variance explained by each of the 13 principal components (dimensions) extracted through FAMD. This plot assists in determining how many components to retain for subsequent analysis

FAMD identified 13 principal components (PCs) with eigenvalues ≥ 1, which together accounted for 74.91% of the total variance. The associated scree plot (Fig. 1) visualized the eigenvalues of these components. The first component explained the largest proportion of variance (23.93%) and had the highest eigenvalue (9.6), followed by the second component. As shown in Fig. 1, the slopes of the first two components were notably steeper, indicating that they captured a larger share of the data's overall structure. Based on the eigenvalue criterion, we retained 13 components for further analysis.

**Fig. 2:**
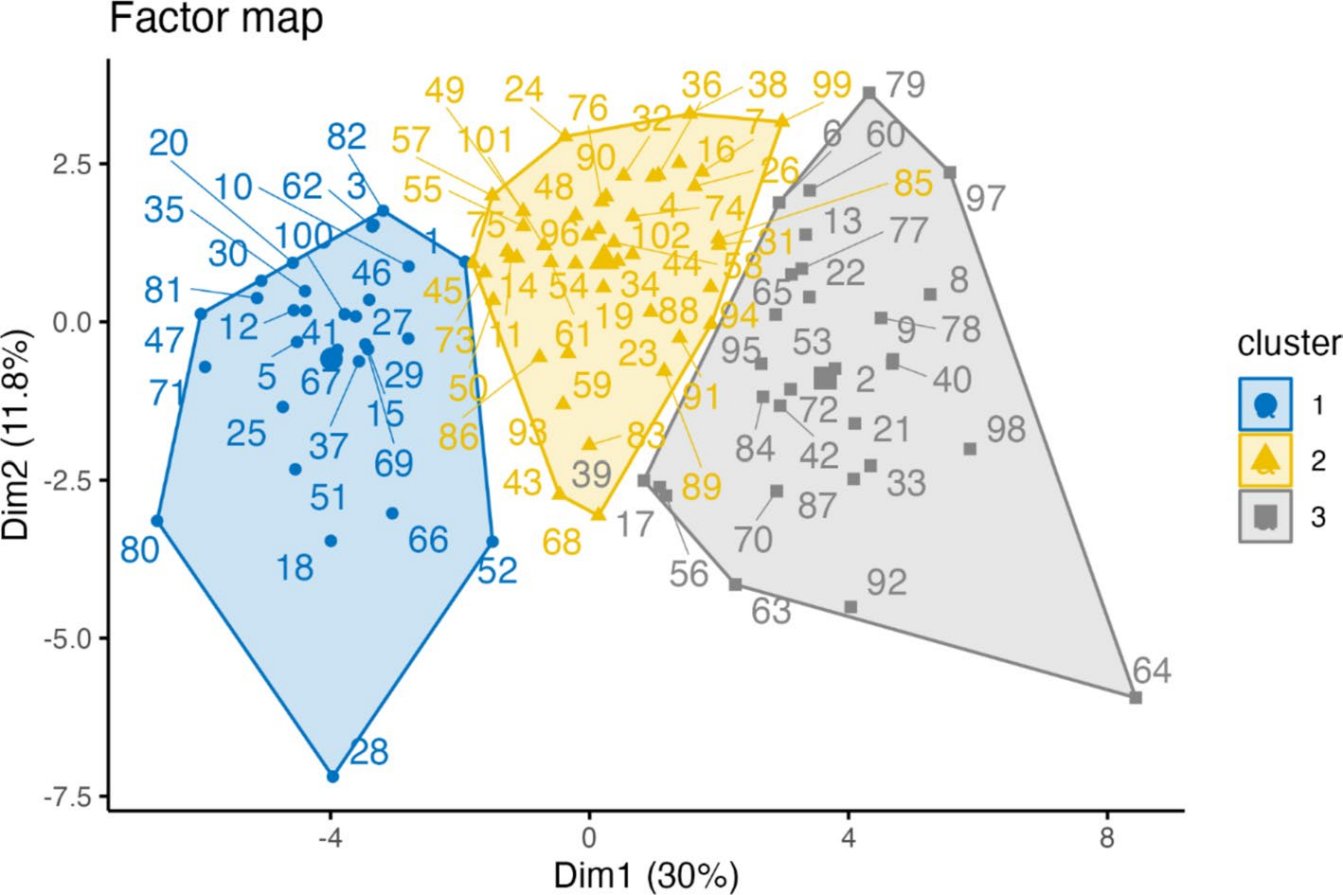
Factor map showing individual observations projected onto the first two principal dimensions (Dim 1 and Dim 2) derived from FAMD. Dimension 1 explains 30% of the variance and Dimension 2 explains 11.8%, together accounting for 41.8% of the total variance. Observations are colored and shaped according to cluster affiliation derived from hierarchical clustering

Hierarchical cluster analysis supported a three-cluster solution as a meaningful way to differentiate participants based on gender dysphoria, social support, general psychopathology, body image, childhood trauma, personality functioning, and identity development. Both statistically and in terms of distinct cluster characteristics, the three-cluster solution provided the best fit for the data, minimizing overlap between groups (Fig. 2). We chose the label “distress” to describe the three-cluster solution, categorizing participants into “Low-Distress,” “Moderate-Distress,” and “High-Distress” groups based on increasing levels of distress and impairments across the assessed domains.

### Characteristics of the Three-Cluster Solution

Table 2 provides an overview of the distribution of participants across the three clusters and contains descriptive statistics, significance levels, and effect sizes for the assessed variables. While the “High-Distress” cluster consisted of slightly older adolescents and a slightly higher proportion of natal males compared to the other clusters, these differences were not statistically significant. There were also no significant differences in the self-reported onset of gender dysphoria between the clusters.

**Table 2:** Descriptive statistics for the three clusters

Measure	Cluster							
	Low-distress		Moderate-distress		High-distress		*p*	*d*
Age in years at assessment (M, SD)	16.10^a^*	1.61	15.88^a^	1.24	16.58^a^	1.48	0.130	0.21
Natal sex							0.475	0.12
Female	24	82.8%	31	70.5%	21	72.4%		
Male	5	17.2%	13	29.5%	8	27.6%		
Onset of gender dysphoria							0.874	0.05
Early	16	55.2%	22	50.0%	16	55.2%		
Late	13	44.8%	22	50.0%	13	44.8%		
Utrecht Gender Dysphoria Scale (UGDS) (M, SD)	54.52^a^	10.45	56.30^a^	6.67	56.41^a^	4.32	0.537	0.11
Gender Identity/Gender Dysphoria Questionnaire (GIDYQ-AA) (M, SD)	2.32^a^	0.55	2.12^ab^	0.26	2.06^b^	0.23	0.017	0.29
Social support								
Support from others	2	6.9%	4	9.1%	6	20.7%	0.203	0.18
No support from others	27	93.1%	40	90.9%	23	79.3%		
Parental support	4.17^a^	1.26	4.30^a^	0.93	2.90^b^	1.52	< 0.0001	0.51
Openness with parents	4.21^a^	1.15	3.82^a^	1.24	2.72^b^	1.41	< 0.0001	0.47
Parental understanding and acceptance	4.10^a^	1.21	3.73^a^	1.06	2.48^b^	1.35	< 0.0001	0.55
Problems due to gender identity	2.17^a^	1.0	2.64^a^	1.22	3.38^b^	1.29	< 0.0001	0.39
Youth Self-Report (YSR)								
Anxious/depressed	53.90^a^	4.39	81.21^b^	13.29	87.48^c^	9.62	< 0.0001	1.34
Withdrawn/depressed	58.72^a^	8.65	78.11^b^	11.92	83.31^b^	11.29	< 0.0001	0.92
Somatic complaints	53.14^a^	4.27	64.68^b^	11.40	79.83^c^	15.58	< 0.0001	0.90
Social problems	55.31^a^	5.97	73.04^b^	10.76	78.79^b^	12.39	< 0.0001	0.93
Thought problems	70.28^a^	13.33	90.55^b^	10.90	98.76^c^	4.13	< 0.0001	1.10
Attention problems	58.14^a^	8.99	65.71^b^	9.60	76.86^c^	8.97	< 0.0001	0.78
Aggressive behavior	52.97^a^	4.32	56.27^b^	5.38	62.97^c^	6.00	< 0.0001	0.74
Rule breaking behavior	53.41^a^	8.07	57.71^a^	8.25	67.83^b^	11.49	< 0.0001	0.62
FBeK (M, SD)								
Attractiveness and self-confidence (reverse-scored)	1.75^a^	0.18	1.88^b^	0.13	1.91^b^	0.14	< 0.0001	0.43
Accentuation of physical appearance	1.55^a^	0.19	1.54^a^	0.19	1.44^a^	0.14	0.032	0.27
Insecurity/concern (reverse-scored)	1.69^a^	0.19	1.42^b^	0.18	1.30^c^	0.17	< 0.0001	0.85
Physical-sexual discomfort	1.17^a^	0.21	1.08^a^	0.28	1.14^a^	0.29	0.312	0.15
BIS overall body dissatisfaction (M, SD)	3.12^a^	0.64	3.49^b^	0.51	3.63^b^	0.38	< 0.0001	0.39
Childhood Trauma Questionnaire (CTQ) (M, SD)								
Emotional abuse	8.31^a^	4.34	10.39^a^	5.00	16.28^b^	4.02	< 0.0001	0.70
Physical abuse	6.07^a^	3.21	5.21^a^	1.07	8.24^b^	4.40	< 0.0001	0.43
Sexual abuse	5.35^a^	1.32	5.39^a^	1.63	9.48^b^	6.24	< 0.0001	0.53
Emotional neglect	10.10^a^	5.54	10.61^a^	4.30	17.41^b^	4.02	< 0.0001	0.70
Physical neglect	6.52^a^	3.73	6.52^a^	2.03	9.17^b^	3.35	< 0.0001	0.41
Levels of Personality Functioning (LoPF-Q) (M, SD)								
Identity	47.41^a^	6.47	63.86^b^	6.79	71.59^c^	7.85	< 0.0001	1.36
Self-direction	47.90^a^	7.16	64.61^b^	8.01	73.07^c^	7.01	< 0.0001	1.31
Empathy	40.93^a^	5.67	51.95^b^	10.69	61.52^c^	10.39	< 0.0001	0.84
Intimacy	49.38^a^	6.32	66.91^b^	11.84	72.24^b^	8.82	< 0.0001	0.96
Identity Diffusion (AIDA) (M, SD)	45.90^a^	5.93	62.55^b^	7.37	71.48^c^	7.38	< 0.0001	1.43

*p* = statistical significance, *d* = effect size Cohen’s *d* (continuous) and Cramers V (categorical data) of the difference between “Low-Distress”, “Moderate-Distress” and “High-Distress” group: *d* > 0.20 small, > 0.50 medium, > 0.80 large; Items on social support were analyzed individually. Four items were rated on 5-point scale and are presented as M (SD); one item was binary (yes/no) and is presented as *n* (%); LoPF, AIDA, and YSR means represent standardized *T*-scores; Responses regarding sexual orientation were aggregated into two categories based on natal sex: (1) same-sex or both-sex attracted, and (2) other orientations (including opposite-sex attraction, ambiguous responses, no attraction, and “other”)

^*^Different letters indicate significantly different groups in multiple testing. Any two groups sharing a letter (e.g., ^a^) were not significantly different. Tukey HSD method was used to adjust *p* values to account for inflation in Type I errors that occur due to multiple testing

#### Gender Dysphoria

Pairwise comparisons for the GIDYQ-AA showed that adolescents in the “Low-Distress” cluster reported significantly less gender dysphoria compared to those in the “High-Distress” cluster, as indicated by higher scores. No significant differences were observed between the “Moderate-Distress” and the other clusters. Gender Dysphoria as measured by the UGDS did not differ significantly across the three clusters.

#### General Psychopathology

The YSR revealed significant differences in general psychopathology across the three clusters. The “High-Distress” cluster consistently showed the most severe symptomatology across both internalizing and externalizing scales. One-sample *t* tests comparing group means with the clinical cut-off of 60 indicated that all *T*-score means in this cluster fell within the clinical range. The “Moderate-Distress” cluster showed *T*-score means within the clinical range for internalizing scales, while means on the externalizing scales were significantly lower than both the “High-Distress" cluster and the clinical cut-off of 60. The “Low-Distress” cluster showed the lowest levels of psychopathological symptoms, with most *T*-score means below the clinical threshold. Notably, all three clusters showed elevated scores on the Thought Problems scale. Pairwise comparisons revealed significant differences between the “Low-Distress” and the “Moderate-Distress” clusters on most scales, while differences between the “Moderate-Distress” and “High-Distress” clusters were more pronounced for externalizing behaviors. Effect sizes ranged from medium to large (Cohen’s *d* = 0.62 to 1.34), with the strongest difference observed for the Anxious/Depressed scale.

#### Support During Role Change

The “High-Distress” cluster reported significantly less perceived social support than the “Low-Distress” and “Moderate-Distress” clusters. These adolescents were less likely to report receiving support during their transition. They also reported receiving less parental support, were less likely to discuss their transition openly with their parents and felt less understood and accepted by their parents. Adolescents in the “High-Distress” cluster also reported significantly more problems related to their gender identity (e.g., with friends or at school). No significant differences were observed between the “Low-Distress” and “Moderate-Distress” clusters on any of these variables.

#### Body Image

Differences in body image were observed across the clusters. As measured by the FBeK, adolescents in the “High-Distress” and “Moderate-Distress” clusters reported significantly lower levels on the Attractiveness/Self-Confidence scale. On the Insecurity/Concern scale, all three clusters differed significantly from each other, with the “High-Distress” cluster reporting the highest levels of insecurity, followed by the “Moderate-Distress” and “Low-Distress” clusters (Cohen’s *d* = 0.85). No significant differences were observed for the Accentuation of Physical Appearance and Physical-Sexual Discomfort scales. Regarding overall body dissatisfaction as measured by the BIS, the “High-Distress” and “Moderate-Distress” clusters reported significantly higher levels of body dissatisfaction than the “Low-Distress” cluster.

#### Prevalence and Severity of Childhood Trauma

The prevalence and severity of childhood trauma, assessed by the CTQ, differed significantly across the three clusters. Adolescents in the “High-Distress” cluster reported the highest levels of childhood trauma exposure across all subscales, including severe to extreme emotional abuse and neglect and moderate to severe sexual abuse. These scores were significantly higher compared to those of both the “Low-Distress” and “Moderate-Distress” clusters (all *p* < 0.001), with effect sizes in the moderate range (Cohen’s *d* = 0.41 to 0.70). The strongest effect size was observed for emotional abuse (Cohen’s *d* = 0.70). No significant differences were found between the “Low-Distress” and “Moderate-Distress” clusters for any of the subscales. Both groups showed low to moderate levels of emotional abuse and/or neglect and little to no trauma exposure on the remaining scales.

#### Levels of Personality Functioning (LoPF-Q) and Identity Development (AIDA)

The LoPF-Q revealed significant differences in personality functioning across the three clusters. Adolescents in the “High-Distress” cluster showed the most severe impairments across all domains, with *T*-scores above the clinical threshold. The “Moderate-Distress” cluster showed clinically significant impairments in the domains Identity, Self-direction, and Intimacy, while Empathy scores remained below the cut-off. In contrast, adolescents in the “Low-Distress” cluster showed no impairments in personality functioning. Pairwise comparisons revealed significant differences between all three clusters (all *p* < 0.001) with large effect sizes (Cohen’s *d* = 0.86 to 1.36), particularly in the self-related domains.

Identity development, as measured by the AIDA, indicated significant differences across the clusters. Adolescents in the “High-Distress” cluster showed pronounced identity diffusion, with *T*-scores well above the clinical cut-off of 60. The “Moderate-Distress” cluster also showed clinically significant impairment in identity development, whereas the “Low-Distress” cluster scored below the clinical threshold. Pairwise comparisons revealed a large effect size (Cohen’s *d* = 1.43).

#### Additional Analyses

While sexual orientation was not included in the cluster analysis, we examined whether cluster assignment was associated with sexual orientation in an exploratory post hoc analysis. No statistically significant association was observed (see Supplementary Table S2).

## Discussion

The purpose of this study was to examine the psychopathological characteristics of a clinical sample of gender dysphoric adolescents seeking treatment at a specialized gender identity service and to identify distinct subgroups using hierarchical cluster analysis. The majority of the sample consisted of natal females, which is consistent with broader international trends in recent years, where the majority of minors presenting to gender identity services with gender dysphoria are natal females (Aitken et al., 2015; Taylor et al., 2024). We examined whether recalled onset of gender dysphoria was associated with natal sex or cluster assignment but did not find any significant associations.

Using hierarchical cluster analysis, we identified three distinct subgroups in our data based on patterns in their levels of gender dysphoria, body image problems, social support, general psychopathology, prevalence and severity of childhood trauma, personality functioning and identity development. The distribution of adolescents across clusters according to natal sex was roughly proportional to their representation in the sample. The first cluster (“Low-Distress”) had the lowest levels of psychopathological symptoms on all measures and reported high levels of social support. The second cluster (“Moderate-Distress”) showed more severe impairments across most dimensions, reporting higher levels of internalizing symptoms, impaired personality functioning and identity development, greater body dissatisfaction, and low to moderate levels of emotional neglect and emotional abuse. However, social support was as high as in the “Low-Distress” cluster. The third cluster (“High-Distress”) was characterized by the highest levels of internalizing and externalizing symptoms, greatest body dissatisfaction, the most severe exposure to childhood trauma, pronounced impairments in all domains of personality functioning and identity integration, and the lowest levels of social support.

Levels of gender dysphoria as measured by the GIDYQ-AA were clinically elevated across all clusters, but pairwise comparisons showed that the “High-Distress” cluster reported significantly higher levels than the “Low-Distress” cluster, while the “Moderate-Distress” cluster overlapped with both. Thus, higher levels of gender dysphoria were associated with greater overall psychopathological impairment in our sample. Further research is needed to better understand the potential mechanisms linking gender dysphoria and psychopathology and to address directionality and possible interactions, which could inform both clinical interventions and theoretical models.

Body image problems, as measured by the BIS and FBeK, were pronounced in all clusters. However, notable differences emerged between clusters on specific subscales. The largest difference was observed for the Insecurity/Concern subscale of the FBeK, which reflects a perceived lack of control over one's body. Adolescence is a critical period for integrating bodily changes associated with sexual maturation into a cohesive sense of self. For adolescents with gender dysphoria, this developmental task can be particularly challenging, as these bodily changes often exacerbate feelings of incongruence and discomfort. Our findings of significant differences between the clusters highlight the need for a nuanced clinical perspective on body dissatisfaction and its interaction with psychopathology in other domains. Scores were most elevated in the “High-Distress” cluster, consistent with their overall distress and heightened gender dysphoria. This finding could also be interpreted in the context of early traumatic experiences, which were significantly more prevalent in the “High-Distress” cluster and have been linked to dissociated body states (Schore, 2003). For some adolescents in this cluster, it may be reasonable to assume that that a disturbed body image is at least partially trauma-related and should be addressed first with trauma-informed clinical approaches.

General psychopathology (YSR) increased across the three clusters, with the “High-Distress” cluster showing the highest levels of symptoms on all subscales, followed by the “Moderate-Distress” cluster. In contrast, adolescents in the “Low-Distress” cluster had almost exclusively subclinical scores, highlighting their comparatively better psychosocial adjustment. Notably, scores on the Thought Problems scale were elevated across all three clusters, indicating a shared symptomatology. Items on this scale assess cognitive and perceptual disturbances such as obsessive thoughts and altered sensory perceptions. Previous research (VanderLaan et al., 2015) suggests that some items on the YSR such as “I can't stop thinking about certain things” or “I have thoughts others might find strange” may capture gender-related preoccupations rather than pathological perceptual disturbances. Such gender-related rumination might have artificially inflated scores on the Thought Problems scale. Some of these items in the YSR allow respondents to add comments that might offer a better understanding of the content of their thoughts. Since such comments were not included in our analysis, we were unable to address the specific content, and our results should therefore be interpreted with caution.

Only adolescents in the “High-Distress” cluster scored above clinical thresholds for externalizing symptoms, consistent with their marked impairment in personality functioning and exposure to childhood trauma. These adolescents also scored highest on internalizing scales, highlighting the need for targeted interventions that address the overall psychopathology in this subgroup.

Adolescents in the “High-Distress” cluster reported significantly lower levels of perceived social support than those in the other two clusters. This finding is consistent with previous research linking low social support to increased psychopathology in gender-diverse youth (Durwood et al., 2021). However, it is important to consider that adolescents with more severe psychopathology may also perceive their social environment as less supportive. The significant psychopathological impairment observed in the “Moderate-Distress” cluster despite high levels of social support suggests that the relationship between mental health and social support for gender dysphoric youth is complex and likely influenced by other psychological, developmental, and environmental factors. One such factor may be exposure to childhood trauma. In this regard, the low levels of social support in the “High-Distress” cluster may reflect pervasive family dysfunction, which is consistent with the high rates of reported childhood trauma, particularly emotional neglect and abuse. While the source of trauma cannot be determined, it is clinically plausible that these experiences may have occurred within primary attachment relationships. Thus, their lack of support is likely not specific to gender incongruence but rather part of a broader pattern of deficient parental attunement and care.

Adolescence is a critical period for identity formation and the consolidation of a coherent sense of self. Early attachment experiences significantly impact on the course of this developmental period, and emerging evidence suggests a link between early relational trauma and impaired personality functioning and identity diffusion in adolescence (Gander et al., 2020, 2025; Penner et al., 2019). When faced with unresolved trauma from earlier developmental stages, adolescence holds the potential for disintegration and the manifestation of significant psychopathology (Fonagy et al., 2002; Schore, 2003). Consistent with this, adolescents in the “High-Distress” cluster showed the most pronounced impairments in both interpersonal (empathy, intimacy) and self-related (identity, self-direction) domains of personality functioning, alongside pronounced identity diffusion and substantial exposure to childhood trauma.

While links between childhood trauma, personality pathology, and identity diffusion are well established (Gander et al., 2020, 2025; Jaffee, 2017), little is known about how these factors shape the experiences of adolescents with gender dysphoria and how they may interact. Recent studies of detransitioners, individuals who have stopped or reversed their gender transition process, suggest that gender dysphoria may emerge in the context of exposure to traumatic experiences and broader mental health impairments (Littman, 2021; Littman et al., 2024; Vandenbussche, 2022). These retrospective reports underscore the need to improve our understanding of how severe psychopathology and gender dysphoria interact in adolescents in order to develop and provide appropriate treatment options. Our findings, particularly for the “High-Distress” cluster, highlight the necessity of a comprehensive diagnostic process and trauma-informed psychotherapy as key components of clinical care.

One of the most pressing issues in the clinical care of adolescents with gender dysphoria is diagnostic assessment and identifying the characteristics that most reliably indicate a persistent transgender identity. This has significant implications for access to medical interventions for minors, for which increasingly strict indication criteria are being demanded (Cass, 2024). The present study aimed to differentiate a clinical sample based on psychopathological variables and identified three clusters with distinct distress profiles within the data. Adolescents in the “High-Distress” cluster exhibited significant psychopathology, showed signs of personality pathology and identity diffusion, reported high levels of childhood trauma experiences and poor social support. Adolescents in the “Moderate-Distress” cluster, on the other hand, reported more social support, less childhood trauma, and showed less severe but still moderate psychopathology overall. Finally, adolescents in the “Low-Distress” cluster showed the lowest levels of psychopathological symptoms and appeared to be the most psychosocially integrated. These differences highlight the need for differentiated diagnostic and therapeutic approaches. In particular, the high rates of childhood trauma and severe impairments in personality functioning and identity integration for some adolescents in this cohort highlighted the need to consider these crucial developmental factors when assessing the needs of gender dysphoric adolescents, as these variables have received very limited research attention despite their clinical relevance.

By identifying these constellations of characteristics, we provided a practical subgroup differentiation and proposed a distress-based typology that needs to be replicated in future research. This framework could provide a foundation for future research aimed at improving our understanding of how different psychopathological traits interact with gender dysphoria. If substantiated by future research, such a typology could inform longitudinal on developmental trajectories of adolescents with gender dysphoria and help identify factors that influence persistence and desistance. Ultimately, this would facilitate the development of prognostic models and support individualized clinical decision-making.

### Limitations

The present findings should be considered in light of several limitations. First, it should be noted that the single clinical cohort studied here limits generalizability to both clinically referred adolescents with gender dysphoria and to adolescents with gender dysphoria in general. Furthermore, the study was based on a cross-sectional design, which means that no causal conclusions can be drawn about the relationships between the variables studied, particularly between gender dysphoria and other psychopathological distress variables.

Regarding the instruments used, it should be noted that they were based solely on self-report, which makes them susceptible to biases such as social desirability or recall bias. Future studies could support this common practice by incorporating parent and expert ratings. In particular, parents' reports could have provided valuable insights and supplemented the information provided by young people, offering a more comprehensive clinical understanding. All instruments used were validated measures with solid psychometric properties, with the exception of the measure of social support, for which we used single items because no validated measure was available. This limitation should be taken into account when interpreting the results, and future research should use a validated measure of social support whenever possible. Most of the questionnaires demonstrated high internal consistencies in this study. However, some subscales showed lower reliability, in particular the Physical-Sexual Discomfort and Accentuation of Physical Appearance scales of the FBeK (α = 0.29 to 0.58) and the male version of the GIDYQ-AA (α = 0.52). The respective results should therefore be interpreted with caution, and future studies may use alternative instruments. In addition, the depth and number of measures vary across constructs. For example, personality functions such as identity were assessed with two different instruments, whereas social support was assessed using single items. Although the dimension reduction approach (FAMD) should have mitigated possible imbalances by prioritizing the most informative components, a more balanced selection of variables should be pursued in future studies. It should also be noted that the selection of instruments was limited, reflecting the range of instruments available in our clinical assessment. Thus, important variables such as neurodevelopmental conditions (e.g., autism or ADHD) were not included in our study and should be incorporated in future research.

We acknowledge that the relatively small sample size in our FAMD and clustering analyses imposes limitations, including reduced statistical power, potential overfitting, and limited generalizability (Hastie et al., 2009; Jolliffe, 2002). In addition, hierarchical clustering may be sensitive to outliers and non-homogeneous distributions. To address these limitations, all continuous variables were standardized, and only components explaining a meaningful proportion of variance were retained. Individual factor maps were examined to identify potential outliers. We assessed the contribution of FAMD dimensions to the clustering solution and analyzed variable distributions across clusters to evaluate coherence and distinctiveness (Pagès, 2004). While the findings are exploratory and should be interpreted with caution, we believe they provide valuable insights into patterns of psychological distress in adolescents with gender dysphoria.

Given the exploratory approach of this study, the cluster solution presented here needs to be replicated in future studies before more definitive conclusions can be drawn. To substantiate the statistical approach presented here, it would be useful to validate the differences between the clusters using independent variables that were not included in the clustering. Furthermore, while the present study focused on subgroup differentiation, future research should also further explore shared patterns across all clusters, such as body image disturbance and psychopathological impairment.

### Conclusion

This study provided a data-driven empirical approach to identifying distinct subgroups in a clinical cohort of adolescents with gender dysphoria based on psychopathological variables using hierarchical cluster analysis. By introducing a distress-based typology, this study highlighted the heterogeneity of this population and underscored the need for individualized treatment approaches. This study provided rare data on childhood trauma, levels of personality functioning, and identity development in a clinical sample of gender dysphoric adolescents, variables that have received limited attention in previous research despite their clinical relevance. The pronounced psychopathology observed in the “High-Distress” cluster, including severe childhood trauma and impaired personality functioning and identity integration, raised important questions about its interaction with gender dysphoria and highlighted the need for careful clinical consideration. Future research should aim to replicate these findings in order to validate the proposed distress typology and make it applicable to future research. Ultimately, this approach could contribute to future research on developmental trajectories and inform the evidence base for individualized treatment approaches tailored to the diverse needs of gender dysphoric adolescents.

## Supplementary Information

Below is the link to the electronic supplementary material.

Supplementary file1 (DOCX 17 KB)

Supplementary file2 (DOCX 13 KB)

Supplementary file3 (PDF 182 KB)

## Data Availability

This clinical data is not publicly available.

## References

[CR1] Achenbach, T. M. (1991). *Manual for the Youth Self-Report and 1991 profile*. University of Vermont.

[CR2] Aitken, M., Steensma, T. D., Blanchard, R., VanderLaan, D. P., Wood, H., Fuentes, A., Spegg, C., Wasserman, L., Ames, M., Fitzsimmons, C. L., Leef, J. H., Lishak, V., Reim, E., Takagi, A., Vinik, J., Wreford, J., Cohen-Kettenis, P. T., de Vries, A. L. C., Kreukels, B. P. C., & Zucker, K. J. (2015). Evidence for an altered sex ratio in clinic-referred adolescents with gender dysphoria. *Journal of Sexual Medicine,**12*(3), 756–763. 10.1111/jsm.12817 25612159 10.1111/jsm.12817

[CR3] American Psychiatric Association. (2013). *Diagnostic and statistical manual of mental disorders* (5th ed.). American Psychiatric Press. 10.1176/appi.books.9780890425596

[CR4] Becker, I., Auer, M., Barkmann, C., Fuss, J., Möller, B., Nieder, T. O., Fahrenkrug, S., Hildebrandt, T., & Richter-Appelt, H. (2018). A cross-sectional multicenter study of multidimensional body image in adolescents and adults with gender dysphoria before and after transition-related medical interventions. *Archives of Sexual Behavior,**47*, 2335–2347. 10.1007/s10508-018-1278-4 30088234 10.1007/s10508-018-1278-4

[CR5] Bernstein, D. P., & Fink, L. (1998). *Childhood Trauma Questionnaire: A retrospective self-report manual*. The Psychological Corporation.

[CR6] Biedermann, S. V., Asmuth, J., Schröder, J., Briken, P., Auer, M. K., & Fuss, J. (2021). Childhood adversities are common among trans people and associated with adult depression and suicidality. *Journal of Psychiatric Research,**141*, 318–324. 10.1016/j.jpsychires.2021.07.016 34304035 10.1016/j.jpsychires.2021.07.016

[CR7] Brähler, E., Strauß, B., Hessel, A., & Schumacher, J. (2000). Standardization of the “Fragebogen zur Beurteilung des eigenen Körpers” (FBeK) in a community-based sample of the German population. *Diagnostica,**46*(3), 156–164. 10.1026/0012-1924.46.3.156

[CR8] Brecht, A., Bos, S., Ries, L., Hübner, K., Widenka, P.-M., Winter, S. M., & Calvano, C. (2024). Analyzing body dissatisfaction and gender dysphoria in the context of minority stress among transgender adolescents. *Child and Adolescent Psychiatry and Mental Health,**18*(1), 30. 10.1186/s13034-024-00718-y 38431595 10.1186/s13034-024-00718-y PMC10909265

[CR9] Cass, H. (2024). *Independent review of gender identity services for children and young people: Final report. Cass Review*. Retrieved from https://cass.independent-review.uk/home/publications/final-report/

[CR10] Charrad, M., Ghazzali, N., Boiteau, V., & Niknafs, A. (2014). Nbclust: An R package for determining the relevant number of clusters in a data set. *Journal of Statistical Software,**61*, 1–36. 10.18637/jss.v061.i06

[CR11] Cohen-Kettenis, P. T., & van Goozen, S. H. (1997). Sex reassignment of adolescent transsexuals: A follow-up study. *Journal of the American Academy of Child & Adolescent Psychiatry,**36*(2), 263–271. 10.1097/00004583-199702000-00017 9031580 10.1097/00004583-199702000-00017

[CR12] Deogracias, J. J., Johnson, L. L., Meyer-Bahlburg, H. F., Kessler, S. J., Schober, J. M., & Zucker, K. J. (2007). The Gender Identity/Gender Dysphoria Questionnaire for Adolescents and Adults. *Journal of Sex Research,**44*(4), 370–379. 10.1080/00224490701586730 18321016 10.1080/00224490701586730

[CR13] Döpfner, M., Plück, J. & Kinnen, C. (2014). *Manual deutsche schulalter-formen der child behavior checklist von Thomas M. Achenbach. Elternfragebogen über das verhalten von kindern und jugendlichen. (CBCL/6–18R), lehrerfragebogen über das verhalten von kindern und jugendlichen (TRF/6–18R), Fragebogen für jugendliche (YSR/11–18R).* [Manual for German school-age forms of the Child Behavior Checklist by Thomas M. Achenbach. Parent questionnaire on behavior of children and adolescents (CBCL/6-18R), teacher questionnaire on the behavior of children and adolescents (TRF/6-18R), questionnaire for adolescents (YSR/11-18R)]. Hogrefe.

[CR14] Durwood, L., Eisner, L., Fladeboe, K., Ji, C., Barney, S., McLaughlin, K. A., & Olson, K. R. (2021). Social support and internalizing psychopathology in transgender youth. *Journal of Youth and Adolescence,**50*(5), 841–854. 10.1007/s10964-020-01391-y 33575917 10.1007/s10964-020-01391-y PMC8272454

[CR15] Feil, K., Riedl, D., Böttcher, B., Fuchs, M., Kapelari, K., Gräßer, S., Toth, B., & Lampe, A. (2023). Higher prevalence of adverse childhood experiences in transgender than in cisgender individuals: Results from a single-center observational study. *Journal of Clinical Medicine,**12*(13), 4501. 10.3390/jcm12134501 37445536 10.3390/jcm12134501 PMC10342728

[CR16] Fonagy, P., Gergely, G., Jurist, E. L., & Target, M. (2002). *Affect regulation, mentalization, and the development of the self*. Other Press.

[CR17] Galupo, M. P., & Pulice-Farrow, L. (2020). Subjective ratings of gender dysphoria scales by transgender individuals. *Archives of Sexual Behavior,**49*(2), 479–488. 10.1007/s10508-019-01575-1 31559520 10.1007/s10508-019-01556-2

[CR18] Gander, M., Buchheim, A., Bock, A., Steppan, M., Sevecke, K., & Goth, K. (2020). Unresolved attachment mediates the relationship between childhood trauma and impaired personality functioning in adolescence. *Journal of Personality Disorders,**34*(Supplement B), 84–103. 10.1521/pedi_2020_34_468 31990614 10.1521/pedi_2020_34_468

[CR19] Gander, M., Buchheim, A., Kohlböck, G., & Sevecke, K. (2025). Unresolved attachment and identity diffusion in adolescence. *Development and Psychopathology,**37*, 429–438. 10.1017/S0954579424000014 38305076 10.1017/S0954579424000014

[CR20] Giovanardi, G., Vitelli, R., Maggiora Vergano, C., Fortunato, A., Chianura, L., Lingiardi, V., & Speranza, A. M. (2018). Attachment patterns and complex trauma in a sample of adults diagnosed with gender dysphoria. *Frontiers in Psychology,**9*, 60. 10.3389/fpsyg.2018.00060 29449822 10.3389/fpsyg.2018.00060 PMC5799708

[CR21] Goth, K., Birkhölzer, M., & Schmeck, K. (2018). Assessment of personality functioning in adolescents with the LoPF–Q 12–18 self-report questionnaire. *Journal of Personality Assessment,**100*(6), 680–690. 10.1080/00223891.2018.1489258 30907712 10.1080/00223891.2018.1489258

[CR22] Goth, K., Foelsch, P., Schlüter-Müller, S., Birkhölzer, M., Jung, E., Pick, O., & Schmeck, K. (2012). Assessment of identity development and identity diffusion in adolescence–theoretical basis and psychometric properties of the self-report questionnaire AIDA. *Child and Adolescent Psychiatry and Mental Health,**6*, 27. 10.1186/1753-2000-6-27 22812911 10.1186/1753-2000-6-27 PMC3485126

[CR23] Haid-Stecher, N., Fuchs, M., Ortner, N., & Sevecke, K. (2020). TransIdentity–identity development among adolescent trans*people. *Praxis der Kinderpsychologie und Kinderpsychiatrie,**69*(6), 541–553. 10.13109/prkk.2020.69.6.541 32988299 10.13109/prkk.2020.69.6.541

[CR24] Hastie, T., Tibshirani, R., & Friedman, J. (2009). *The elements of statistical learning: Data mining, inference, and prediction* (2nd ed.). Springer.

[CR25] Herrmann, L., Fahrenkrug, S., Bindt, C., & Becker-Hebly, I. (2023). Gender experiences of transgender youth: How changeable is the gender experience of binary vs. nonbinary identifying transgender youth and what factors are involved? *Zeitschrift für Kinder und Jugendpsychiatrie und Psychotherapie,**52*(1), 12–29. 10.1024/1422-4917/a000957 37947191 10.1024/1422-4917/a000957

[CR100] Husson, F., Josse, J., & Pagès, J. (2010). *Principal component methods hierarchical clustering partitional clustering: Why would we need to choose for visualizing data?* [Technical Report]. http://www.sthda.com/english/upload/hcpc_husson_josse.pdf

[CR26] Jaffee, S. R. (2017). Child maltreatment and risk for psychopathology in childhood and adulthood. *Annual Review of Clinical Psychology,**13*(1), 525–551. 10.1146/annurev-clinpsy-032816-045005 28375720 10.1146/annurev-clinpsy-032816-045005

[CR27] Jolliffe, I. T. (2002). *Principal component analysis*. Springer.

[CR28] Kaltiala-Heino, R., Bergman, H., Työläjärvi, M., & Frisén, L. (2018). Gender dysphoria in adolescence: Current perspectives. *Adolescent Health, Medicine and Therapeutics*. 10.2147/AHMT.S135432 29535563 10.2147/AHMT.S135432 PMC5841333

[CR29] Karvonen, M., Goth, K., Eloranta, S. J., & Kaltiala, R. (2022). Identity integration in adolescents with features of gender dysphoria compared to adolescents in general population. *Frontiers in Psychiatry,**13*, 848282. 10.3389/fpsyt.2022.848282 35757222 10.3389/fpsyt.2022.848282 PMC9218247

[CR30] Kaufman, L., & Rousseeuw, P. J. (1990). *Finding groups in data: An introduction to cluster analysis*. Wiley.

[CR31] Kernberg, O. (1986). *Severe personality disorders: Psychotherapeutic strategies*. Yale University Press.

[CR32] Klinitzke, G., Romppel, M., Häuser, W., Brähler, E., & Glaesmer, H. (2011). The German version of the Childhood Trauma Questionnaire (CTQ): Psychometric characteristics in a representative sample of the general population. *Psychotherapie, Psychosomatik, Medizinische Psychologie,**62*(2), 47–51. 10.1055/s-0031-1295495 22203470 10.1055/s-0031-1295495

[CR33] Kozlowska, K., Chudleigh, C., McClure, G., Maguire, A. M., & Ambler, G. R. (2021a). Attachment patterns in children and adolescents with gender dysphoria. *Frontiers in Psychology, 11*. 10.3389/fpsyg.2020.582688 33510668 10.3389/fpsyg.2020.582688 PMC7835132

[CR34] Kozlowska, K., McClure, G., Chudleigh, C., Maguire, A. M., Gessler, D., Scher, S., & Ambler, G. R. (2021b). Australian children and adolescents with gender dysphoria: Clinical presentations and challenges experienced by a multidisciplinary team and gender service. *Human Systems,**1*(1), 70–95. 10.1177/26344041211010777

[CR35] Lawrence, A. A. (2010). Sexual orientation versus age of onset as bases for typologies (subtypes) of gender identity disorder in adolescents and adults. *Archives of Sexual Behavior,**39*, 514–545. 20140487 10.1007/s10508-009-9594-3

[CR36] Lê, S., Josse, J., & Husson, F. (2008). Factominer: An R package for multivariate analysis. *Journal of Statistical Software,**25*, 1–18. 10.18637/jss.v025.i01

[CR37] Lemma, A. (2021). *Transgender identities: A contemporary introduction*. Routledge.

[CR38] Levine, S. B., & Abbruzzese, E. (2023). Current concerns about gender-affirming therapy in adolescents. *Current Sexual Health Reports,**15*(2), 113–123. 10.1007/s11930-023-00358-x

[CR39] Lindgren, T. W., & Pauly, I. B. (1975). A body image scale for evaluating transsexuals. *Archives of Sexual Behavior,**4*, 639–656. 10.1007/BF01544272 1212093 10.1007/BF01544272

[CR40] Littman, L. (2021). Individuals treated for gender dysphoria with medical and/or surgical transition who subsequently detransitioned: A survey of 100 detransitioners. *Archives of Sexual Behavior,**50*(8), 3353–3369. 10.1007/s10508-021-02163-w 34665380 10.1007/s10508-021-02163-w PMC8604821

[CR41] Littman, L., O’Malley, S., Kerschner, H., & Bailey, J. M. (2024). Detransition and desistance among previously trans-identified young adults. *Archives of Sexual Behavior,**53*(1), 57–76. 10.1007/s10508-023-02716-1 38038854 10.1007/s10508-023-02716-1 PMC10794437

[CR42] Mahmood, M. S. (2021). *Factor analysis of mixed data. Towards data science*. Retrieved from: https://towardsdatascience.com/factor-analysis-of-mixed-data-5ad5ce98663c/

[CR43] Murtagh, F., & Contreras, P. (2012). Algorithms for hierarchical clustering: An overview. *Wiley Interdisciplinary Reviews: Data Mining and Knowledge Discovery,**2*(1), 86–97. 10.1002/widm.53

[CR44] Pagès, J. (2004). Analyse factorielle de données mixtes. *Revue de Statistique Appliquée,**52*(4), 93–111.

[CR101] Penner, F., Gambin, M., & Sharp, C. (2019). Childhood maltreatment and identity diffusion among inpatient adolescents: The role of reflective function. *Journal of Adolescence, 76*, 65–74. 31472427 10.1016/j.adolescence.2019.08.002

[CR45] RStudio Team. (2022). *RStudio: Integrated development for R (version 2022.07.2)* [computer software]. Posit.

[CR46] Schneider, C., Cerwenka, S., Nieder, T. O., Briken, P., Cohen-Kettenis, P. T., De Cuypere, G., Haraldsen, I. R., Kreukels, B. P. C., & Richter-Appelt, H. (2016). Measuring gender dysphoria: A multicenter examination and comparison of the Utrecht Gender Dysphoria Scale and the Gender Identity/Gender Dysphoria Questionnaire for Adolescents and Adults. *Archives of Sexual Behavior,**45*, 551–558. 10.1007/s10508-016-0702-x 26883025 10.1007/s10508-016-0702-x

[CR47] Schore, A. (2003). *Affect regulation and the repair of the self*. W.W. Norton & Company.

[CR48] Singh, D., Deogracias, J. J., Johnson, L. L., Bradley, S. J., Kibblewhite, S. J., Owen-Anderson, A., & Zucker, K. J. (2010). The Gender Identity/Gender Dysphoria Questionnaire for Adolescents and Adults: Further validity evidence. *Journal of Sex Research,**47*(1), 49–58. 10.1080/00224490902954341 19396705 10.1080/00224490902898728

[CR49] Steensma, T. D., Kreukels, B. P., Jurgensen, M., Thyen, U., de Vries, A. L. C., & Cohen-Kettenis, P. T. (2013). The Utrecht Gender Dysphoria Scale: A validation study. In T. D. Steensma (Ed.), *From gender variance to gender dysphoria: Psychosexual development of gender atypical children and adolescents* (pp. 41–56). Vrije Universiteit Amsterdam.

[CR50] Strauß, B., & Richter-Appelt, H. (1996). *Fragebogen zur beurteilung des eigenen körpers (FBeK)* [questionnaire on the assessment of one’s own body]. Hogrefe Verlag für Psychologie.

[CR51] Stübler, M. L., & Becker-Hebly, I. (2019). Sexuelle erfahrungen und sexuelle orientierung von transgender-jugendlichen. *Zeitschrift für Sexualforschung,**32*(1), 5–16. 10.1055/a-0838-8965

[CR52] Taylor, J., Hall, R., Langton, T., Fraser, L., & Hewitt, C. E. (2024). Characteristics of children and adolescents referred to specialist gender services: A systematic review. *Archives of Disease in Childhood,**109*, s3–s11. 10.1136/archdischild-2023-326681 38594046 10.1136/archdischild-2023-326681

[CR53] Thompson, L., Sarovic, D., Wilson, P., Sämfjord, A., & Gillberg, C. (2022a). A PRISMA systematic review of adolescent gender dysphoria literature: (1) epidemiology. *PLoS Global Public Health,**2*(3), e0000245. 10.1371/journal.pgph.0000245 36962334 10.1371/journal.pgph.0000245 PMC10021877

[CR54] Thompson, L., Sarovic, D., Wilson, P., Sämfjord, A., & Gillberg, C. (2022b). A PRISMA systematic review of adolescent gender dysphoria literature: (2) mental health. *PLoS Global Public Health,**2*(5), e0000426. 10.1371/journal.pgph.0000426 36962230 10.1371/journal.pgph.0000426 PMC10021389

[CR55] Vandenbussche, E. (2022). Detransition-related needs and support: A cross-sectional online survey. *Journal of Homosexuality,**69*(9), 1602–1620. 10.1080/00918369.2021.1919479 33929297 10.1080/00918369.2021.1919479

[CR56] VanderLaan, D. P., Postema, L., Wood, H., Singh, D., Fantus, S., Hyun, J., Leef, J., Bradley, S. J., & Zucker, K. J. (2015). Do children with gender dysphoria have intense/obsessional interests? *Journal of Sex Research,**52*(2), 213–219. 10.1080/00224499.2013.860073 24558954 10.1080/00224499.2013.860073

[CR57] World Health Organization. (2019). *International statistical classification of diseases and related health problems* (11th ed.). World Health Organization. 10.2471/BLT.25.294650 PMC13531092 42683092

[CR58] Zucker, K. J. (2019). Adolescents with gender dysphoria: Reflections on some contemporary clinical and research issues. *Archives of Sexual Behavior,**48*, 1983–1992. 10.1007/s10508-019-01518-8 31321594 10.1007/s10508-019-01518-8

