# Supplementary material for: Distress Profiles of Adolescents with Gender Dysphoria: A Cluster Analysis Approach
Source: Arch Sex Behav. 2025 Aug 20;54(8):3095–109. doi: 10.1007/s10508-025-03221-3 (PMC12484269; doi:10.1007/s10508-025-03221-3)
Supplement: Supplementary file 1 — Supplementary file1 (DOCX 17 KB) [file 10508_2025_3221_MOESM1_ESM.docx]

**Supplementary Table S1**

Sociodemographic characteristics and YSR scores of included vs. excluded participants

|  | Excluded  *N* = 49 | Included  *N* = 102 | *p* |
| --- | --- | --- | --- |
| Age in years (mean, SD) | 15.8 (1.3) | 16.1 (1.4) | 0.151 |
| Natal sex |  |  | 0.493 |
| Female | 39 (79.6%) | 76 (74.5%) |  |
| Male | 10 (20.4%) | 26 (25.5%) |  |
| Living with mother |  |  |  |
| Biological mother | 41 (85.4%) | 93 (94.9%) | 0.050 |
| Not living with Biological mother (i.e. Adoptive mother, Foster mother, Stepmother; Replacement mother) | 7 (14.6%) | 5 (5.1%) |  |
| Living with father |  |  | 0.233 |
| Biological father | 31 (66.0%) | 55 (55.6%) |  |
| Not living with Biological mother (i.e. Adoptive father, foster father, Step Father; Replacement father) | 16 (34.0%) | 44 (44.4%) |  |
| Marital status of parents |  |  |  |
| Separated/divorced | 24 (50.0%) | 41 (40.2%) | 0.661 |
| Deceased | 0 (0%) | 2 (2.0%) |  |
| Never lived together | 0 (0%) | 2 (2.0%) |  |
| Married partnership | 22 (50.0%) | 57 (55.9%) |  |
| Place of residence: |  |  |  |
| City >100,000 inhabitants | 1 (5.0%) | 21 (20.6%) | 0.247 |
| Rural | 12 (60.0%) | 49 (48.0%) |  |
| Small Town | 7 (35.0%) | 32 (31.4%) |  |
| Occupation |  |  |  |
| Apprenticeship/work | 6 (12.2%) | 15 (14.7%) | 0.463 |
| Unemployed or looking for a job | 11 (22.4%) | 12 (11.8%) |  |
| Attending school | 31 (63.3%) | 70 (68.6%) |  |
| School drop out | 0 (0%) | 1 (1.0%) |  |
| Vocational training | 1 (2.0%) | 4 (3.9%) |  |
| Onset of gender dysphoria |  |  | 0.208 |
| Early (≤ 10 years) | 43 (56.6%) | 11 (42.3%) |  |
| Late (> 10 years) | 33 (43.4%) | 15 (57.7%) |  |
| Sexual Orientation |  |  |  |
| Same-sex or both-sex attracted | 10 (55.6%) | 70 (71.4%) | 0.181 |
| Other | 8 (34.2%) | 28 (28.6%) |  |
| Youth Self-Report (YSR): |  |  |  |
| Anxious/Depressed | 76.3 (13.1) | 70.7 (13.0) | 0.096 |
| Withdrawn/Depressed | 76.2 (13.3) | 71.7 (13.5) | 0.185 |
| Somatic Complaints | 69.6 (6.9) | 62.9 (10.3) | 0.009 |
| Social Problems | 69.2 (10.7) | 65.6 (8.8) | 0.111 |
| Thought Problems | 81.4 (9.5) | 76.9 (10.4) | 0.086 |
| Attention Problems | 67.5 (11.2) | 66.0 (11.3) | 0.599 |
| Aggressive Behavior | 56.8 (5.5) | 56.4 (7.3) | 0.849 |
| Rule Breaking Behavior | 61.8 (10.6) | 59.3 (8.2) | 0.260 |

*Note*. N varies slightly for included and more substantially for excluded participants due to partial nonresponse. For example, YSR data were available for only 18 participants who were excluded from the study. YSR means represent standardized T-scores.
Responses regarding sexual orientation were aggregated into two categories based on natal sex: (1) same-sex or both-sex attracted, and (2) other orientations (including opposite-sex attraction, ambiguous responses, no attraction, and “other”).
