# Supplementary material for: Distress Profiles of Adolescents with Gender Dysphoria: A Cluster Analysis Approach
Source: Arch Sex Behav. 2025 Aug 20;54(8):3095–109. doi: 10.1007/s10508-025-03221-3 (PMC12484269; doi:10.1007/s10508-025-03221-3)
Supplement: Supplementary file 2 — Supplementary file2 (DOCX 13 KB) [file 10508_2025_3221_MOESM2_ESM.docx]

**Supplementary Table S2**

Distribution of sexual orientation across the three clusters.

|  | Low-Distress  *N* = 29 | Moderate-Distress  *N* = 47 | High-Distress  *N=23* | *p* |
| --- | --- | --- | --- | --- |
| Sexual Orientation: |  |  |  | 0.607 |
| Same-sex or both-sex attracted | 22 (75.9 %) | 31 (66.0 %) | 17 (73.9%) |  |
| Other | 7 (24.1 %) | 16 (34.0 %) | 6 (26.1%) |  |

*Note*. Responses regarding sexual orientation were aggregated into two categories based on natal sex: (1) same-sex or both-sex attracted, and (2) other orientations (including opposite-sex attraction, ambiguous responses, no attraction, and “other”).
