# Supplementary material for: Distress Profiles of Adolescents with Gender Dysphoria: A Cluster Analysis Approach
Source: Arch Sex Behav. 2025 Aug 20;54(8):3095–109. doi: 10.1007/s10508-025-03221-3 (PMC12484269; doi:10.1007/s10508-025-03221-3)
Supplement: Supplementary file 3 — Supplementary file3 (PDF 182 KB) [file 10508_2025_3221_MOESM3_ESM.pdf]

Correlations	Age at As	Support of par	Talking openly	Understood an	Any probler	UGDS	GIDY	Attractiver	Accentuat	Insecurity Con	Sexual Dissatis	BIS Overall Bo	YSR Anxious	YSR Withdrav	YSR Somati	YSR Social P	YSR Thought	YSR Attentio	YSR Aggres	YSR Rule Bre	CTQ Emotic	CTQ Physica	CTQ Sexual	CTQ Emotio	CTQ Physica	LoPF_Identi	LoPF Self D	LoPF Empat	LoPF Intima	AIDA Diffus
Age at Assessment	--																													
Support of parents during role change	-0,159	--																												
Talking openly to parents about role change	0,197*	0,755**	--																											
Understood and accepted as I am	0,196*	0,804**	0,841**	--																										
Any problems reported with role change	-0,104	-0,087	-0,012	-0,094	--																									
UGDS	0,110	0,066	0,032	0,020	0,063	--																								
GIDY	-0,014	-0,062	-0,084	-0,032	-0,178	0,484**	--																							
Attractivensess/self-confidence	-0,041	0,031	-0,120	-0,022	0,053	0,248*	0,261**	--																						
Accentuation of body appearance	-0,022	0,104	0,122	0,111	-0,168	-0,090	0,088	-0,008	--																					
Insecurity Concern	-0,015	0,037	0,137	0,150	0,219*	-0,155	0,307**	0,440**	0,224*	--																				
Sexual Dissatisfaction	0,081	-0,159	-0,085	-0,099	0,003	0,249*	0,059	-0,105	0,055	0,090	--																			
BIS Overall Body Dissatisfaction	0,061	-0,056	-0,115	-0,033	0,092	0,310**	0,299**	0,638**	-0,138	0,426**	-0,111	--																		
YSR Anxious/Depressed	0,087	-0,136	0,260**	0,228*	0,276**	0,000	-0,150	0,459**	0,242*	0,647**	-0,161	0,414**	--																	
YSR Withdrawn	0,186	-0,141	0,277**	0,198*	0,056	0,056	-0,040	0,463**	-0,078	0,455**	0,301**	0,357**	0,760**	--																
YSR Somatic Complaints	0,037	-0,164	0,258**	0,297**	0,303**	0,045	0,273**	0,239*	0,206*	0,516**	-0,064	0,239*	0,499**	0,400**	--															
YSR Social Problems	-0,020	-0,121	0,272**	0,269**	0,331**	0,012	-0,128	0,367**	-0,066	0,491**	-0,188	0,291**	0,671**	0,564**	0,455**	--														
YSR Thought Problems	0,150	-0,133	0,250*	0,283**	0,243*	0,055	0,248*	0,332**	-0,131	0,538**	-0,100	0,299**	0,749**	0,599**	0,522**	0,606**	--													
YSR Attention Problems	0,011	-0,090	-0,159	-0,187	0,112	0,116	0,264**	0,206*	-0,109	0,440**	0,019	0,182	0,460**	0,353**	0,501**	0,398**	0,623**	--												
YSR Aggressive Behaviors	0,098	0,246*	-0,162	0,240*	0,269**	0,054	0,202*	0,153	-0,110	0,237*	0,072	0,128	0,367**	0,206*	0,383**	0,389**	0,472**	0,468**	--											
YSR Rule Breaking Behavior	0,008	-0,086	-0,172	-0,176	0,118	0,075	0,255**	0,158	-0,109	0,421**	0,031	0,151	0,420**	0,323**	0,499**	0,385**	0,583**	0,981**	0,482**	--										
CTQ Emotional Abuse	0,122	0,339**	0,407**	0,396**	0,235*	0,074	0,208*	0,137	0,203*	0,318**	0,015	0,015	0,321**	0,211*	0,375**	0,290**	0,342**	0,303**	0,370**	0,322**	--									
CTQ Physical Maltreatment	0,117	0,403**	0,355**	0,360**	0,074	-0,095	-0,083	0,069	-0,115	-0,129	0,132	-0,074	0,008	-0,040	0,198*	0,136	0,155	0,130	0,317**	0,134	0,462**	--								
CTQ Sexual Abuse	0,105	0,371**	0,253*	0,344**	0,149	-0,078	-0,068	0,150	-0,110	0,199*	0,016	0,067	0,237*	0,184	0,210*	0,316**	0,255**	0,141	0,380**	0,142	0,270**	0,500**	--							
CTQ Emotional Neglect	0,199*	0,439**	0,512**	0,531**	0,171	0,003	-0,083	0,046	-0,021	-0,186	0,115	-0,054	0,132	0,099	0,321**	0,225*	0,236*	0,255**	0,263**	0,280**	0,743**	0,569**	0,335**	--						
CTQ Physical Neglect	0,088	0,428**	0,424**	0,462**	0,064	-0,012	-0,156	0,022	0,039	-0,110	0,121	-0,068	0,057	-0,010	0,201*	0,240*	0,120	0,156	0,240*	0,177	0,401**	0,555**	0,334**	0,672**	--					
LoPF_Identity	0,128	-0,135	0,251*	0,229*	0,272**	0,089	0,249*	0,448**	-0,153	0,648**	-0,067	0,398**	0,780**	0,646**	0,503**	0,627**	0,660**	0,542**	0,467**	0,519**	0,430**	0,089	0,263**	0,251*	0,147	--				
LoPF Self Direction	0,071	-0,139	0,252*	0,238*	0,196*	0,068	-0,190	0,462**	0,262**	0,648**	-0,040	0,447**	0,795**	0,651**	0,500**	0,613**	0,675**	0,567**	0,471**	0,534**	0,391**	0,067	0,217*	0,212*	0,096	0,866**	--			
LoPF Empathy	0,028	-0,120	0,199*	0,241*	0,310**	-0,010	-0,181	0,257**	-0,121	0,321**	0,227*	0,223*	0,466**	0,541**	0,417**	0,596**	0,457**	0,404**	0,431**	0,402**	0,256**	0,143	0,283**	0,250*	0,189	0,614**	0,570**	--		
LoPF Intimacy	0,096	-0,073	0,205*	-0,158	0,171	0,089	-0,138	0,415**	-0,037	0,448**	0,257**	0,301**	0,628**	0,744**	0,315**	0,654**	0,504**	0,339**	0,253*	0,323**	0,318**	0,108	0,230*	0,221*	0,166	0,673**	0,625**	0,658**	--	
AIDA Diffusion	0,031	-0,133	0,239*	0,231*	0,327**	0,037	0,252*	0,431**	0,222*	0,633**	-0,064	0,394**	0,789**	0,615**	0,526**	0,661**	0,674**	0,544**	0,492**	0,527**	0,424**	0,116	0,275**	0,281**	0,177	0,950**	0,872**	0,644**	0,690**	--
*. Correlation is significant at the 0.05 level (2-tailed).																														
**. Correlation is significant at the 0.01 level (2-tailed).																														
